# Effect of *hfq* (Host Factor for RNA Phase Q_*β*_ Replicase) on Environmental Tolerance of *Escherichia coli* Under Simulated Microgravity

**DOI:** 10.1155/ijm/9150440

**Published:** 2025-09-10

**Authors:** Huaxian Li, Peijun Han, Ya Li, Xinxin Li, Yong Liu, Wenlan Wang

**Affiliations:** ^1^Department of Aerospace Hygiene, School of Aerospace Medicine, Air Force Medical University, Xi'an, Shaanxi Province, China; ^2^Department of Pediatrics, Tangdu Hospital, Fourth Military Medical University, Xi'an, Shaanxi Province, China

**Keywords:** environmental tolerance, *Escherichia coli*, *hfq*, simulated microgravity

## Abstract

During space flights, opportunistic pathogens carried by astronauts may change their biological characteristics under weightless conditions, potentially affecting the health of astronauts. Simulated microgravity (SMG) has been reported to influence the environmental tolerance, which is related to *Escherichia coli*'s environmental tolerance. But the underlying mechanisms remain unclear. Host factor for RNA phage Q_*β*_ replicase (*hfq*) is an RNA molecular chaperone protein found in bacteria. In this study, we discovered that *hfq* expression in *E. coli* increases under SMG through transcriptome sequencing analysis. We knocked out hfq in *E. coli* to establish a *Δhfq*–*E. coli* strain to investigate the effect of *hfq* on the environmental tolerance of *E. coli* under SMG, and observed changes in Coproporphyrinogen III dehydrogenase (*hemN*) and RNA polymerase sigma factor (*rpoS*), which are related to the environmental tolerance of *E. coli*. Results showed that the growth level of *Δhfq*–*E. coli* was significantly lower than that of the wild-type strain under SMG. *E. coli* enhances its tolerance to oxidative stress through the *hfq*–*hemN* pathway under SMG. After SMG exposure, *E. coli*'s tolerance to high temperatures and acidic environments changed, but these changes were not mediated by the *hfq*–*rpoS* pathway; instead, they may act synergistically. This study provides a foundation for preventing and controlling opportunistic pathogenic bacterial infections during aerospace missions.

## 1. Introduction

Aerospace environmental factors include weightlessness, radiation, low pressure, hypoxia, and others. Since special environmental factors such as low pressure and hypoxia, solar ultraviolet radiation, and other unique factors can be mitigated by spacecraft barriers, weightlessness is one of the main factors affecting astronauts' health in current aerospace activities and is also a focus of space medical research. During space activities, astronauts may carry some opportunistic pathogens into the space station. Under the influence of weightlessness, the infectivity, immune characteristics, pathogenicity, and drug resistance of these opportunistic pathogens may change accordingly [1, 2]. At the same time, some studies have found that the aerospace environment can also weaken astronauts' immune function [3]. Therefore, opportunistic pathogens may pose a threat to astronauts' health in space stations.

*Escherichia coli* (*E. coli*) is the most common opportunistic pathogen in humans [4–6]. Its colonization in astronauts provides it with the opportunity to enter spacecraft and be affected by weightlessness. Some studies have reported that under weightlessness or simulated microgravity (SMG), *E. coli* can undergo a series of changes, such as increased growth levels, drug resistance, and virulence, which can increase its threat to health [7, 8]. Host factor for RNA phage Q_*β*_ replicase (Hfq) is an RNA molecular chaperone protein found in *E. coli*, *Pseudomonas aeruginosa*, and other bacteria [9–11]. Studies have shown that *hfq* can affect the growth level, environmental tolerance, and drug resistance of bacteria [12]. Morita and Aiba and Tsui et al. [13, 14] have found that the absence of *hfq* can inhibit the growth of *E. coli*. Chao and Vogel [12] found that *hfq* can promote bacterial cells' ability to resist oxidative stress and acidic environmental stress.

Coproporphyrinogen III oxidase is an enzyme involved in heme biosynthesis. Previous studies [15, 16] have shown that there are two different types of Coproporphyrinogen III oxidase: one for aerobic conditions as an oxygen-dependent oxidase (HemF) and one for anaerobic conditions as an oxygen-independent dehydrogenase (HemN). Azzouzi et al. [17] found that the copper-targeted tetrapyrrole biosynthesis pathway can affect the activity of bacterial oxidase and bacterial photosynthesis at the level of Coproporphyrinogen III oxidase HemN. Rohaun et al. [18] found that hydrogen peroxide does not reduce the activity of HemN in *E. coli*, indicating that HemN is resistant to environmental stress caused by hydrogen peroxide. Previous studies [19] have shown that *P. aeruginosa* can enhance its environmental stress tolerance under simulated weightlessness conditions through the AlgU → Hfq → Anr → HemN pathway, but this pathway has not been studied in *E. coli*.

RpoS is a sigma factor (*σ*^s^) in the stable growth stage of *E. coli*. As a major regulatory factor, it can activate multiple genes involved in most stress responses [20]. It is the main regulatory factor for most stress responses in *E. coli* and other gamma-deformed bacteria [21]. RpoS can resist high osmotic pressure as well as environmental stress from low and high temperatures [22]. Soper et al. [23] found that the translation of RpoS is completely dependent on Hfq, and the Hfq binding site on mRNA (RpoS) plays a key role in regulating its translation process, and Hfq contributes to the interaction between sRNA and mRNA. DsrA is a small RNA that effectively regulates the translation of rpoS and requires the involvement of the RNA chaperone Hfq [24]. Meanwhile, *hfq* can regulate the metabolism of *E. coli*, positively regulate RpoS [25], and promote the binding between RpoS and DsrA to regulate bacterial response to environmental stress [26, 27]. Previous studies [14, 28] have shown that the interaction between Hfq and sRNA in *rpoS* mRNA regulation is crucial for the stress response of *E. coli* and *P. aeruginosa*.

However, the impact and mechanism of *hfq* on the environmental tolerance of *E. coli* under SMG are still unclear, and there have been no reports on whether *hemN* and *rpoS* are involved in this process. Therefore, in this study, we used the 3D gravity microgravity environment simulator to establish an SMG model of *E. coli* and knocked out *hfq* to establish the *hfq*-deficient strains of *E. coli* strain (*Δhfq*–*E. coli*) in order to explore the effect and mechanism of *hfq* on the environmental tolerance of *E. coli* in the SMG environment.

## 2. Material and Methods

### 2.1. Instruments and Equipment

The instruments and equipment used are as follows: gravity control system (Space Bio Laboratories, Japan), constant temperature incubator (DH-500AS, Beijing Kewei Yongxing Instrument Co. Ltd., China), constant temperature oscillator (T42-D, Suzhou Peiying Experimental Equipment Co. Ltd., China), constant temperature oscillation incubator (YJDY-140C, Shanghai Iridium Crystal Technology Co. Ltd., China), biosafety cabinet (NU-543-400S, Nuaire, United States), biosafety centrifuge (Microfuge 16, Bio-Rad, United States), ultramicro UV spectrophotometer (NanoDrop One, Thermo Scientific, United States), PCR instrument (T100 Thermal Cycler, Bio-Rad, United States), real-time fluorescence quantitative PCR instrument (LC480, Roche, Switzerland), MicroPulser Electroporator (1652100, Bio-Rad, United States), Qubit 2.0 fluorescence meter (Q32866, Invitrogen, United States), electrophoresis apparatus (DYY-11, Beijing Liuyi Instrument Factory, China), biological electrophoretic image analysis (FR-980A, Shanghai Furi Technology Co. Ltd., China), biomicroscope (BA210 Digital, McDotti Industrial Group Co. Ltd., China), critical point dryer (K850, Quorum, United Kingdom), ion sputtering apparatus (Smart Coater, JEOL, Japan), and scanning electron microscope (SEM) (JSM-IT700HR, Smart Coater, JEOL, Japan).

### 2.2. Reagents

The reagents used are as follows: tryptone, yeast extract, NaCl (Shanghai Shenggong Bioengineering Co. Ltd., China), 3% glutaraldehyde (AR grade) and anhydrous ethanol (China National Pharmaceutical Group Chemical Reagent Co. Ltd.), osmic acid (Beijing Zhongjing Science and Technology Co. Ltd.), tannins (Shanghai McLean Biochemical Technology Co. Ltd., China), RNA preservation solution and total RNA extraction (Shanghai Shenggong Bioengineering Co. Ltd., China), Qubit 2.0 RNA detection kit, Qubit RNA detection kit, and Qubit DNA detection kit (Life Technologies, United States), Ribo-off rRNA depletion kit (bacteria) VAHTS stranded mRNA-seq V2, Library Prep Kit for Illumina, and VAHTS DNA Clean Beads (Nanjing Nuoweizan Biotechnology Co. Ltd., China), MightyScript First Chain cDNA Synthesis Master Mix (Shanghai Shenggong Bioengineering Co. Ltd., China), and QuantiNova SYBR Green PCR Kit (Qiagen, Germany).

### 2.3. *E. coli* Strain and Culture Medium

*E. coli* (CICC 10389) was purchased from the China Center of Industrial Culture Collection (CICC). LB liquid culture medium and LB solid culture medium were used for *E. coli* cultivation:

LB liquid culture medium: 10 g of tryptone, 5 g of yeast extract, and 5 g of NaCl, dissolved in 1000 mL of double-distilled water, autoclaved at 121°C for 30 min, stored at 4°C, and adjusted to pH around 7.0.

LB solid culture medium: 10 g of tryptone, 5 g of yeast extract, 5 g of NaCl, and 20 g of agar powder, dissolved in 1000 mL of double-distilled water, autoclaved at 121°C for 30 min, then inverted onto a flat plate, stored at 4°C, and adjusted to pH around 7.0.

### 2.4. Cultivation of *E. coli* in SMG Environment and Normal Gravity (NG) Environment

**Figure 1:**
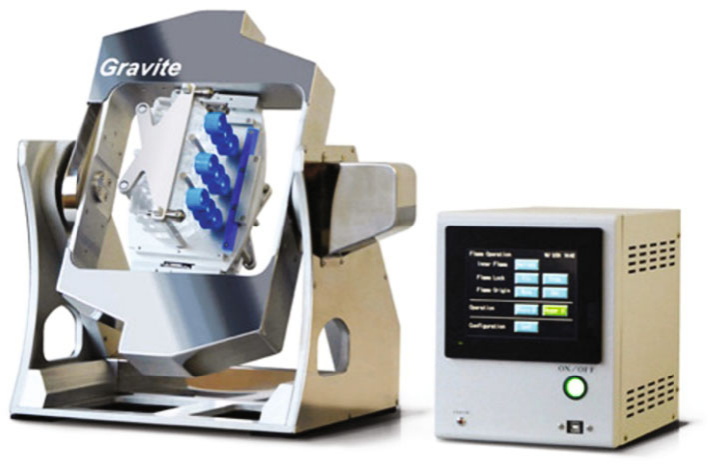
Gravite gravity control system.

The SMG environment is established using the Gravite control system [29, 30] (Figure 1). This system is a multidirectional G-force generator that can simultaneously control the rotation of two axes, that is, rotate in three-dimensional space. This unique feature allows for the cancellation of accumulated gravity vectors at the center of the device, creating a 10^−3^ g microgravity environment similar to that of the International Space Station (ISS). The culture bottles were placed on the sample rack to simulate the microgravity environment for *E. coli* cultivation. Bacteria in the NG environment were placed in a constant temperature oscillator and oscillated horizontally for cultivation in the culture bottle. In both cultivation environments, the preserved monoclonal *E. coli* strains were isolated and inoculated on LB plates with an inoculation ring stripe, incubated at 37°C for 12 h, and single colonies were selected and inoculated in a shaking tube containing 5 mL of LB liquid medium at 37°C, 200 r/min, and activated overnight. The activated bacterial solution was inoculated into a culture bottle filled with fresh LB liquid medium (volume about 73 mL) at a volume ratio of 1:500, and the culture solution was slowly replenished to fill the culture bottle, thereby expelling bubbles from the culture bottle.

### 2.5. Transcriptome Sequencing

#### 2.5.1. Extraction and Quality Inspection of RNA

**Figure 2:**
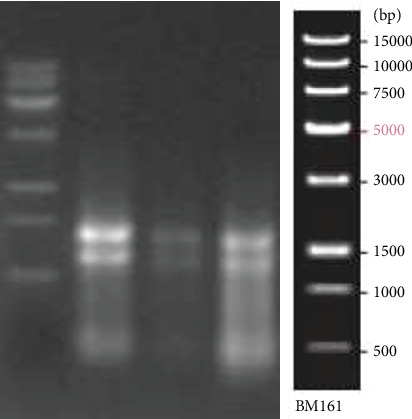
Gel electrophoresis test results (sampling order: marker, YJ, SPY, gravity; Note: marker is a DNA marker, which is only used to detect genomic contamination and does not represent RNA band size).

1. Using the total RNA extraction kit, the sample was lysed and placed at room temperature for 5–10 min to completely separate nucleoprotein from nucleic acid.
2. Add 200 *μ*L of chloroform to the sample, shake thoroughly, and let it stand at room temperature for 3 min. Centrifuge at 12,000 r/min at 4°C for 10 min.
3. Transfer the supernatant to a clean centrifuge tube, add an equal volume of isopropanol, mix well, and let it stand at room temperature for 20 min. Centrifuge at 12,000 r/min at 4°C for 10 min and discard the supernatant.
4. Add 1 mL of 75% ethanol to wash the precipitate after centrifugation. Centrifuge at 12,000 r/min at 4°C for 3 min and discard the supernatant.
5. Dry the sample at room temperature for 5–10 min. Add 30–50 *μ*L RNase-free ddH_2_O to fully dissolve RNA.
6. Store the extracted RNA at −70°C or use immediately.
7. Qubit 2.0 was used to detect RNA concentration, and agarose gel was used to detect RNA integrity and genome contamination. The quality inspection results show that the sample quality basically meets the quality requirements for library sequencing [31]. The test results are as follows (Table 1 and Figure 2).

#### 2.5.2. Constructing a Prokaryotic RNA Transcriptome Library

This part was entrusted to Shenggong Bioengineering (Shanghai) Co. Ltd. for completion.

#### 2.5.3. Differential Analysis of Gene Expression

TMM (trimmed mean of M-values) was used to standardize the read count data, followed by DEGseq for differential analysis. The screening criteria for genes with significant differences were *p* ≤ 0.05 and |log_2_ fold change| ≥ 1 [32].

#### 2.5.4. GO and KEGG Enrichment Analysis of Key Targets

Using the GO database and KEGG database, clusterProfiler was used for functional enrichment analysis. When *p* < 0.05, it was considered that the function had significant enrichment.

### 2.6. *hfq* Gene Knocking Out in *E. coli*

**Figure 3:**
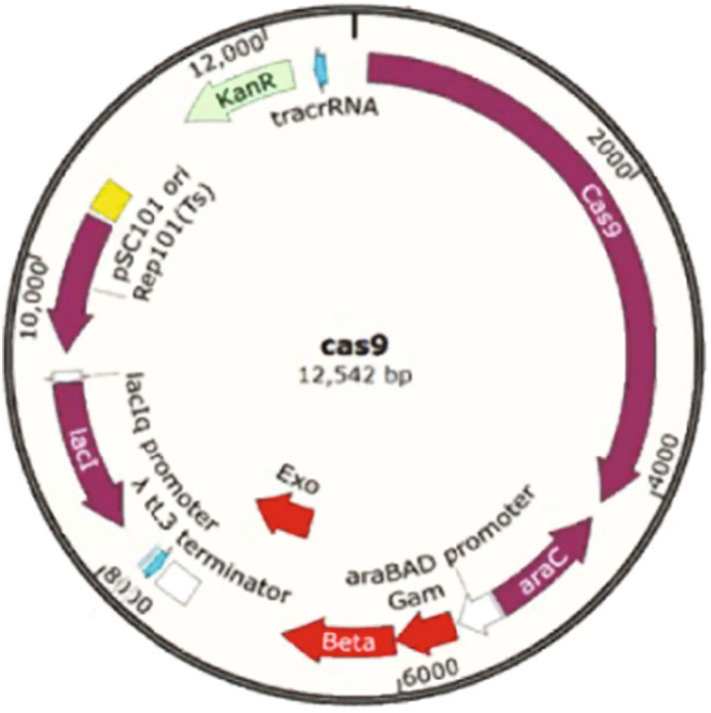
Plasmid expressing Cas9 protein (KanR resistance).

**Figure 4:**
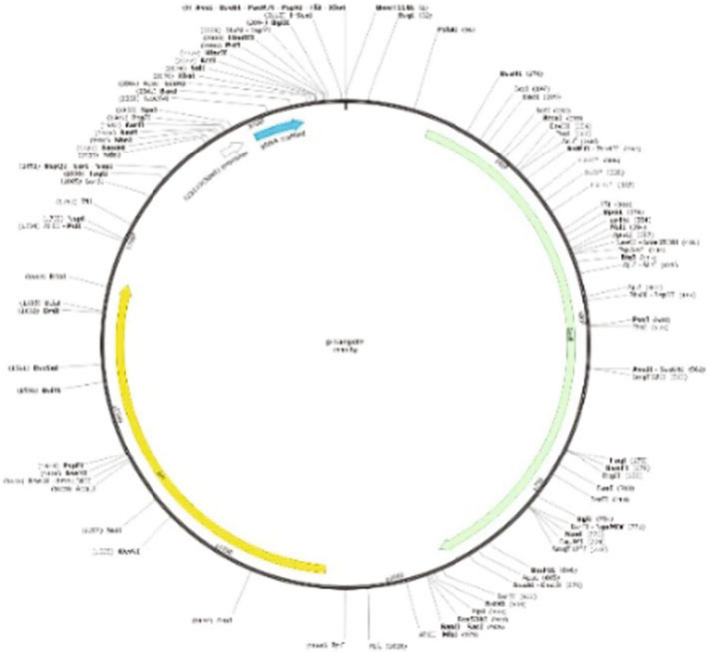
Plasmid and map of p-sgRNA (insertion site is SpeI).

The CRISPR-Cas9 system was used for gene editing of the *E. coli* genome to establish *Δhfq–E. coli*. This system required the synthesis of two plasmids and a homologous recombination template. The bacterial solution of *E. coli* was transferred to 200 mL LB medium at 1% and cultured to OD_600_ of 0.6 and then subpackaged and stored at −80°C. The plasmid expressing the Cas9 protein (KanR resistance) was prepared. The plasmid was from Hangzhou Adcoris Biotechnology Co. Ltd., and the plasmid map is shown in Figure 2. Design the sgRNA (CGACGCAGTGCGTTCAGGAA), and synthesize the p-sgRNA plasmids based on the target sequence that needs to be knocked out (Figure 3). Then, the homologous recombination template was synthesized, and the DNA repair template after cleavage was synthesized. First, electroporate Cas9 into the target strain, and then electroporate p-sgRNA along with the recombinant template into the strain containing the Cas9 plasmid for sequence knockout (Figure 4). The Cas plasmid contains the temperature-sensitive replicon repA101 (Ts), which prevents it from replicating properly at higher temperatures. When bacteria are cultured at a high temperature of 37°C, the repA101 (Ts) replicon cannot function properly, resulting in the plasmid being unable to replicate during cell division and gradually being diluted. The positive monoclonal colonies of *E. coli* strains that expressed the Cas9 protein simultaneously with the p-sgRNA vector and recombinant template were selected for PCR sequencing to verify the knockout results.

### 2.7. Bacterial Grouping

*E. coli* were divided into four groups, including *E. coli* of Gravite simulated weightlessness (ECG) group, *E. coli* of normal gravity (ECN) group, *E. coli* after *hfq* gene knockout under Gravite simulated weightlessness (*Δhfq*-ECG) group, and *E. coli* after *hfq* gene knockout under normal gravity (*Δhfq*-ECN) group. The ECG group and *Δhfq*-ECG group were placed in the Gravite gravity control system at 2 r/min at 37°C. The ECN group and *Δhfq*-ECN group were placed in a thermostatic oscillator at 2 r/min at 37°C. Bacteria were inoculated in a new culture bottle with a volume ratio of 1:500 every 24 h and cultured continuously for 14 days.

### 2.8. Measurement of Growth Curve

After 14 days of continuous culture of *E. coli* in different groups, 10 culture bottles with filter membranes were prepared for each group (to maintain the oxygen supply of bacteria), filled with LB liquid medium, respectively. The cultured bacterial solution was inoculated according to the volume of 1:500. The OD_600_ value of each sample was measured by spectrophotometer every 2 h for a total of 20 h. The test was repeated at least three times for each group.

### 2.9. SEM

Fixation: Bacteria collected by centrifugation should be visibly precipitated from sesame seeds to mung bean size, gently rinsed with PBS, and discarded PBS. Added with 3% glutaraldehyde in the sample, suspended, and fixed;

Postfixation: Wash the bacteria with ultrapure water three times, each time for 10 min. Fixed with 1% osmium acid for 1–2 h, then washed with ultrapure water three times, each time for 10 min;

Dehydration: Alcohol is dehydrated step by step, with a concentration gradient of 30% → 50% → 70% → 90% → 100% (100% concentration is changed three times), each time for 15 min;

Drying: Drop the bacteria onto a cover glass, and place it in a critical point dryer for drying;

Conductive treatment: Attach the cover glass to the sample stage with conductive adhesive, and place it in an ion sputtering machine for gold spraying treatment.

The JSM-IT700HR SEM produced by JEOL was used to capture images of the samples. Each sample was observed in its entirety at low magnification before selecting the area to be observed to capture images and observe its specific morphology.

### 2.10. Determination of Environment Stress Tolerance

According to the survival conditions, common disinfection and sterilization ranges of *E. coli*, acidic environment (pH 3.5), high-temperature environment (50°C), oxidative stress environment (0.003%), and hypertonic environment (2.5 mol/L NaCl) were selected to stimulate the bacteria. The concentration of the cultured bacterial solution was adjusted by OD_600_ to 1.0, and the LB liquid medium with the above conditions and the corresponding blank control samples (LB liquid medium, 37°C) were added according to the volume ratio of 1:100. All the samples were incubated at 37°C for 1 h except for the high-temperature group, which was incubated at 50°C for 1 h. After incubation, the culture medium was diluted with PBS in double ratio, and the concentration of 10^−4^ was selected to be coated on LB solid medium for counting. Each plate was coated with 100 *μ*L bacterial solution, and three plates were coated with each concentration of bacterial solution. The coated solid medium was placed in a constant temperature incubator and incubated at 37°C until the colonies appeared. The survival rate of each sample after stimulation was calculated as (*a*_l_ + *a*_2_ + *a*_3_)/(*b*_1_ + *b*_2_ + *b*_3_). *a*_l_, *a*_2_, and *a*_3_ were the coating count results after incubation for 1 h under stimulation conditions, and *b*_1_, *b*_2_, and *b*_3_ were the coating count results after incubation for 1 h in ordinary LB medium [33, 34]. Each group of tests should be repeated for at least three times.

### 2.11. Real-Time qPCR Experiments

Bacterial RNA was extracted from each group, and the Master Mix Kit MightyScript was used to synthesize the first-strand cDNA for reverse transcription. The extracted RNA was quantified by an ultrafine ultraviolet spectrophotometer. To ensure that every 20 *μ*L reaction system contained 500 ng template RNA, DEPC water was added to 10 *μ*L, and then 10 *μ*L of 2× II M-MLV RT Mix was added to produce a complete reverse transcription system. Reverse transcription was performed by PCR, and the program was set at 25°C for 5 min, 42°C for 30 min, and 85°C for 5 min. RT-qPCR was performed on the reverse-transcribed cDNA, and the preparation system was 1 *μ*L cDNA. Add upstream and downstream primers for 1 *μ*L each, 2× SYBR Green PCR Master Mix 10 *μ*L, and DEPC Water 7 *μ*L. Preheat the PCR amplifier for one cycle at 95°C for 2 min. Forty cycles were amplified, and each cycle lasted for 5 s at 95°C and 10 s at 60°C. After one cycle of dissolution, samples lasted for 5 s at 95°C and for 10 s at 60°C, and then slowly increased to 97°C. Samples were cooled for one cycle for 30 s at 40°C. Each experiment was repeated at least three times. The primer sequence is shown in Table 2.

### 2.12. Statistical Analysis

The data was analyzed using SPSS 26.0 software, using a double sample test, and the results were presented as $\bar{x}\pm s$. When *p* < 0.05, the difference is considered statistically significant.

## 3. Results

### 3.1. *hfq*, *hemN*, *and ropS* of *E. coli* Increased Under SMG

**Figure 5:**
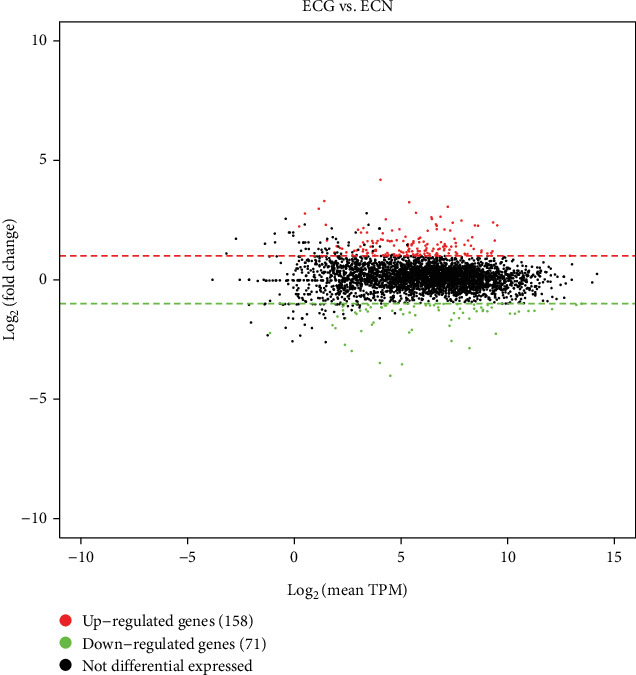
Differential gene expression MA map (ECG vs. ECN).

**Figure 6:**
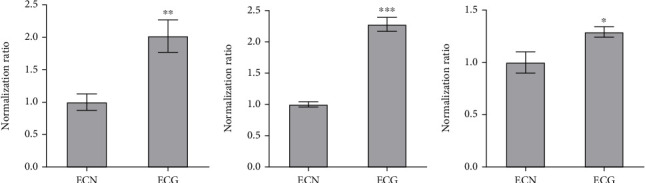
Verify the transcriptome results through RT-qPCR (data are presented as the mean ± SD; *n* = 6; (a) hfq, (b) hemN, (c) rpoS; ⁣^∗^*p* < 0.05,^∗∗^*p* < 0.01,^∗∗∗^*p* < 0.001; ECG vs. ECN).

In transcriptome sequencing analysis, compared with NG, the *E. coli* of the SMG group had 229 significantly differentially expressed genes (*p* ≤ 0.05, |log_2_fold change| ≥ 1), of which 158 genes were upregulated and 71 genes were downregulated (Figure 5). The differentially expressed genes related to environmental stress tolerance were screened by transcriptome sequencing analysis and verified by RT-qPCR, among which the expression of hfq, hemN, and rpoS was increased compared with the NG group (*p* < 0.05) (Table 3 and Figure 6).

### 3.2. *hfq* Had Been Successfully Knocked Out in *Δhfq*–*E. coli*

**Figure 7:**
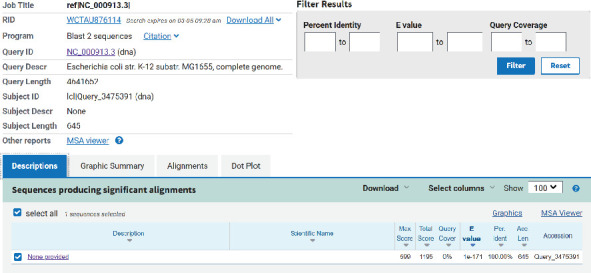
Comparison of two genomes in NCBI database (*Δhfq*–*E. coli* vs. *E coli*).

The expression level of the *hfq* gene was detected through RT-qPCR. The results showed that the expression of *Δhfq*–*E. coli* could not be detected. In addition, this result was validated through DNA sequencing, and the sequence of *Δhfq*–*E. coli* had been uploaded in GenBank (GenBank: PP281333.1). The sequencing results are as follows: CAGGAGACGCTCTACCGTATCAGGTGCATCAGTTCGCCATCGCCCCGGCGAGCCGTGAACTGCTCCATCAACGCATTGAGCAGCGTTTTCATCAGATGTTGGCTTCAGGTTTTGAAGCAGAAGTCCGGGCGCTTTTTGCCCGAGGAGATTTGCATACGGACTTGCCTTCCATTCGTTGCGTGGGTTATCGCCAGATGTGGTCTTACCTTGAAGGCGAAATCTCATACGATGAAATGGTTTATCGAGGTGTTTGCGCCACGAGACAGTTGGCGAAGCGGCAGATAACCTGGCTGCGTGGTTGGGAAGGGGTTCACTGGCTTGACACCTCCAGGAGTTTGAATCTCTGGTCTCTTCCGCCGGTGTCGAAGCATTGCAGGTGATTACCGGTAGCCGTAAAGCGCCGCACCCAAAGTATTTTGTAGGTGAAGGTAAAGCAGTTGAAATTGCGGAAGCTGTCAAAGCGACGGGTGCTTCGGTCGTTCTTTTTGACCATGCCCTGAGCCCGGCGCAAGAGCGTAACCTGGAGCGTTTGTGCGAGTGTCGTGTTATCGACCGCACCGGCCTTATTTTAGATATTTTCGCCCAACGTGCGCGTACCCATGAGGGTAAGTTGCAGGTTGAGCTGGCGCAGCTGCGCCATCTGGC. In addition, comparing the two gene sequences revealed significant differences between them (Figure 7).

### 3.3. *hfq* Played a Positive Regulatory Role in the Growth Level of *E. coli* Under the SMG

**Figure 8:**
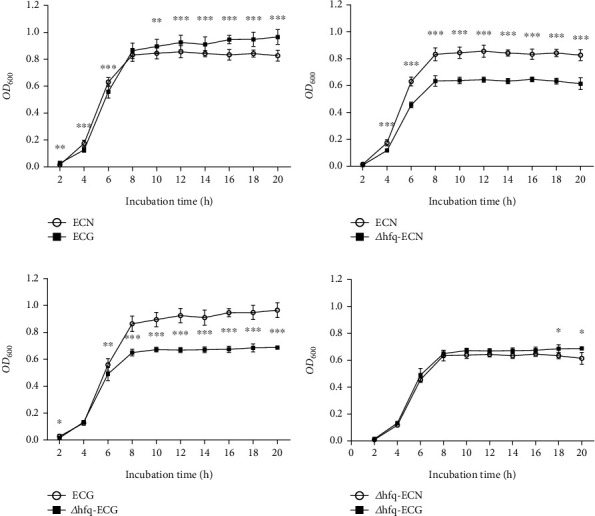
Growth curve trend (data are presented as the mean ± SD; *n* = 6; (a) ECG vs. ECN, (b) *Δhfq*-ECN vs. ECN, (c) *Δhfq*-ECG vs. ECG, (d) *Δhfq*-ECG vs. *Δhfq*-ECN; ⁣^∗^*p* < 0.05,^∗∗^*p* < 0.01,^∗∗∗^*p* < 0.001).

The OD_600_ values of each group were measured after 14 days of continuous culture. The growth level of ECN was higher than that of ECG at the logarithmic stage before 8 h, and the difference was statistically significant (*p* < 0.05). At about 8 h of growth, the growth rate of ECG was higher than that of ECN, and the growth rates of the two groups were in a crossover state, with no statistical significance (*p* > 0.05). In the stable period after 8 h of growth, the OD_600_ of ECG was significantly higher than that of ECN (*p* < 0.05). This indicates that simulating weightlessness conditions promotes the growth of *E. coli* during the stable phase more effectively. However, approaching the stable stage, the growth level of *Δhfq*–*E. coli* strains was significantly lower than that of wild-type *E. coli* (WT *E. coli*) under both the NG and the SMG environments. There was no significant difference between *Δhfq*-ECN and *Δhfq*-ECG. It was indicated that *hfq* played a positive regulatory role in the growth of *E. coli* approaching the stable stage (Figure 8).

### 3.4. Under the SMG Conditions, the Size of *E. coli* Is Not Only Regulated by *hfq*

**Figure 9:**
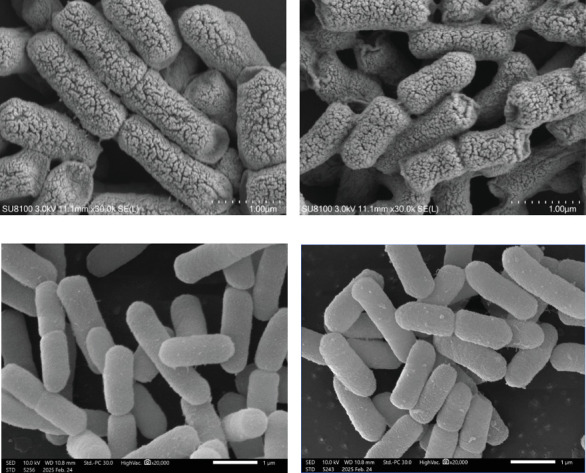
Scanning electron microscopy: (a) ECN, (b) ECG, (c) *Δhfq*-ECN, and (d) *Δhfq*-ECG).

**Figure 10:**
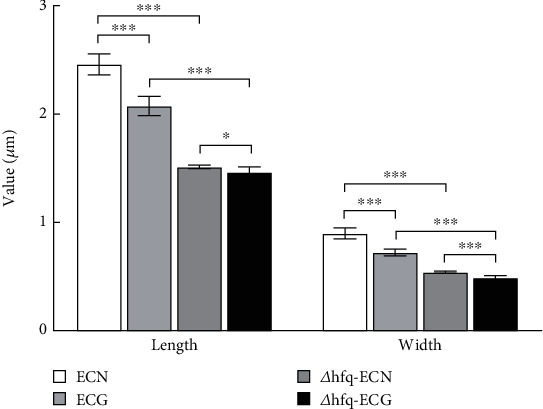
Statistical chart of scanning electron microscopy results (data are presented as the mean ± SD; *n* = 6; ⁣^∗^*p* < 0.05,^∗∗∗^*p* < 0.001).

*E. coli* cultured continuously for 14 days, and the SEM results showed that the morphology of *E. coli* could be reduced under the SMG conditions (Figure 9); at the same time, the morphology of the hfq-deficient strain was significantly smaller than that of the WT *E. coli*. It indicated that *hfq* plays an important role in the morphology and size of *E. coli*. In the two groups of *hfq*-deficient strains, the morphology of *E. coli* under the SMG conditions was also smaller than that of the NG group (Figure 10). Combining the above conclusions, it suggests that under the SMG conditions, the size of *E. coli* is not only regulated by *hfq*, but also that *hfq* is not the main factor affecting the size of *E. coli* under the SMG conditions.

### 3.5. Effect of hfq on Environmental Tolerance of *E. coli* Under the SMG

#### 3.5.1. *hfq* Negatively Regulated the Tolerance of *E. coli* to Acidic Environment

**Figure 11:**
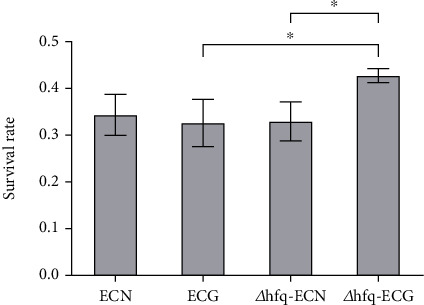
Changes in tolerance to acidic environment (data are presented as the mean ± SD; *n* = 6; ⁣^∗^*p* < 0.05).

After 14 days of continuous culture under the SMG, the tolerance of *E. coli* to the acidic environment had no significant change compared with that of the NG group. The survival rate of *Δhfq*–*E. coli* in a pH 3.5 environment after the SMG was higher than that in the NG group. At the same time, under the SMG, the survival rate of *Δhfq*–*E. coli* strain was higher than that of the WT *E. coli*. These results indicate that the SMG does not affect the tolerance of WT *E. coli* to acidic environments; under the NG, *hfq* has no significant effect on the tolerance of *E. coli* to acidic environments, but the absence of *hfq* enhances the tolerance of *E. coli* to acidic environments under the SMG (Figure 11).

#### 3.5.2. *hfq* Negatively Regulated the Tolerance of *E. coli* to Hypertonic Environment

**Figure 12:**
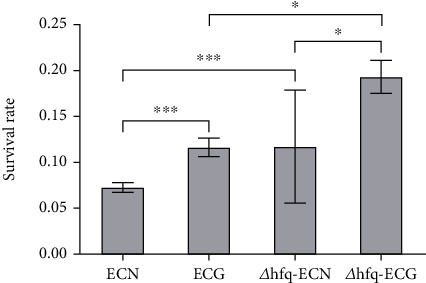
Changes in tolerance to hypertonic environment (data are presented as the mean ± SD; *n* = 6; ⁣^∗^*p* < 0.05,^∗∗∗^*p* < 0.001).

After 14 days of continuous culture under the SMG, the tolerance of *E. coli* to a hypertonic environment which contained 2.5 mol/L (17%) NaCl was significantly increased compared with that in the NG group. After the SMG, the survival rate of *Δhfq*–*E. coli* in 2.5 mol/L (17%) NaCl was higher than that in *Δhfq*-ECN group. Under both the NG and the SMG conditions, the survival rate of *Δhfq*–*E. coli* strain increased compared with that of WT *E. coli*. These results indicated that *hfq* negatively regulated the tolerance of *E. coli* to a hypertonic environment, the tolerance of *E. coli* to a hypertonic environment was affected by the SMG, and the absence of *hfq* enhances the tolerance of *E. coli* to a hypertonic environment (Figure 12).

#### 3.5.3. *hfq* Positively Regulated the Tolerance of *E. coli* to Oxidative Stress Environment

**Figure 13:**
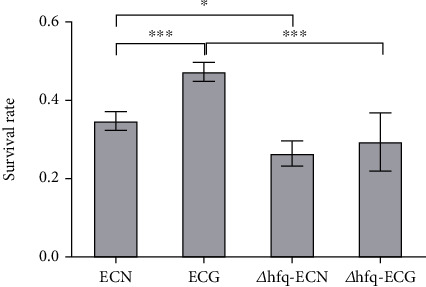
Changes in tolerance to oxidative stress environment (data are presented as the mean ± SD; *n* = 6; ⁣^∗^*p* < 0.05,^∗∗∗^*p* < 0.001).

After 14 days of continuous culture under the SMG, the tolerance of *E. coli* to the oxidative stress environment which contains 0.003% hydrogen peroxide was significantly increased compared with that in the ECN group. Under both NG and the SMG, the survival rate of *Δhfq*–*E. coli* strain was significantly lower than that of WT *E. coli*. After the SMG, the survival rate of *Δhfq*–*E. coli* was not significantly different from that in *Δhfq*-ECN. These results indicated that *hfq* positively regulated the tolerance of *E. coli* to the oxidative stress environment, the tolerance of *E. coli* to oxidative stress environment was affected by the SMG, and the absence of *hfq* weakened the tolerance of *E. coli* to oxidative stress (Figure 13).

#### 3.5.4. The Regulatory Factors of High-Temperature Tolerance of *E. coli* Are Not Only *hfq*

**Figure 14:**
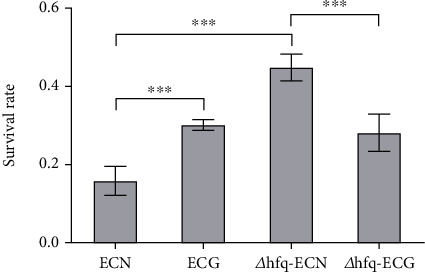
Changes in tolerance to high-temperature environment (data are presented as the mean ± SD; *n* = 6; ⁣^∗∗∗^*p* < 0.001).

After 14 days of continuous culture under the SMG, the tolerance of ECG to high-temperature environment (50°C) was significantly increased compared with that in the ECN group. These results indicated that the SMG conditions can increase the tolerance of *E. coli* to high temperatures. But the survival rate of *Δhfq*-ECN strain was higher than that of the ECN under the NG, and there was no significant difference in survival rate between *Δhfq*-ECG strain and ECG in the SMG. These results suggested that under the NG, *hfq* negatively regulates the high-temperature tolerance of *E. coli*. But, in the *Δhfq*–*E. coli*, the SMG reduced the tolerance of *E. coli* to high temperatures. These conclusions indicated that the impact of *E. coli* on high-temperature tolerance is not only regulated by *hfq* (Figure 14).

### 3.6. Effect of *hfq* on Expression of Tolerance-Related Genes in *E. coli*

#### 3.6.1. Effect of *hfq* on Expression of *hemN*

**Figure 15:**
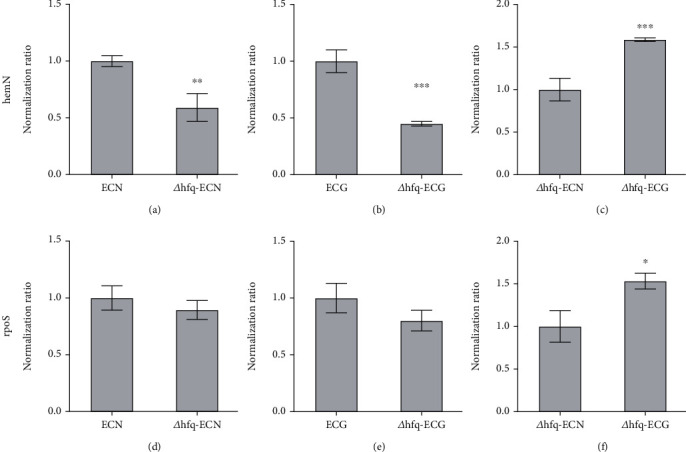
(a–f) Changes in *hemN* and *rpoS* expression (data are presented as the mean ± SD; *n* = 6; ⁣^∗^*p* < 0.05, ⁣^∗∗^*p* < 0.01,^∗∗∗^*p* < 0.001).

The expression of *hemN* in *Δhfq*–*E. coli* strain decreased significantly compared with that of WT *E. coli* under both the NG and the SMG. After the SMG, the expression of *hemN* in both the WT *E. coli* and the *Δhfq*–*E. coli* was increased. These results indicated that *hemN* was positively regulated by *hfq*, and the SMG could increase *hemN* expression (Figures 15a, 15b, and 15c).

#### 3.6.2. Effect of *hfq* on Expression of *rpoS*

For *rpoS*, there was no significant difference between *Δhfq*–*E. coli* and WT *E. coli* under both the NG and the SMG. But the expression of *rpoS* increased in both the WT *E. coli* and the *Δhfq*–*E. coli* after the SMG. These results indicated that the expression of *rpoS* was not regulated by *hfq*, but was affected by the SMG (Figures 15d, 15e, and 15f).

## 4. Discussion

Due to the increasing working hours of astronauts on the space station, the biosafety issues of bacteria carried by astronauts themselves and present in spacecraft should be given more attention. As is well known, *E. coli* is the most common conditioned pathogen and may cause infections, and it had been detected on both the ISS and the Mir space station [36, 37]. In our previous study, we conducted transcriptome sequencing of *E. coli* cultured under SMG and NG and found that *hfq* was closely related to the biological characteristic changes of *E. coli* after SMG [31]. Therefore, studying the effect of *hfq* on the environmental tolerance of *E. coli* under SMG is of great significance for future aerospace biosafety.

*hfq* was first discovered in the RNA replication process of *E. coli* Q_*β*_ phage [38]. As a pleiotropic regulator, *hfq* regulates the stability and translation function of bacteria, especially in posttranscriptional regulation of gene expression [39–41]. Researchers found that the absence of *hfq* could inhibit the growth of *E. coli* [13, 14]. This is consistent with our research results, indicating that *hfq* has a positive regulatory effect on the growth of *E. coli*. At the same time, our study found that after hfq knockout, there was no significant difference in the growth of *E. coli* in the SMG compared to the NG. This suggests that in the SMG environment, the growth of *E. coli* is mostly regulated by *hfq*.

Previous studies have shown that *hfq* is an important factor regulating virulence in *E. coli* [38, 42]. It was also reported that *hfq* could promote the ability of bacteria to resist environmental stress such as oxidative stress and acidity [12, 38]. So we knocked out *hfq* in the *E. coli* by CRISPR-Cas9 method to build *Δhfq*–*E. coli* strain. And we found that compared with the wild strain, the growth level of *Δhfq*–*E. coli* was decreased, the tolerance to oxidative stress was decreased, and the tolerance to acid and hypertonic stress was increased. Although there was no significant difference in the survival rate between *Δhfq*–*E. coli* strain and the wild strain in high temperature under SMG, the tolerance of *E. coli* to high temperature was still affected by SMG. All the results indicated that *hfq* played an important role in the environmental tolerance of *E. coli* under SMG.

*hemN* is a kind of oxygen-independent dehydrogenase and can affect the activity of bacterial oxidase and the photosynthesis of bacteria through the copper-targeted tetrapyrrole biosynthesis pathway [15, 16]. Studies found that *hemN* was resistant to environmental stress caused by hydrogen peroxide [17, 18]. There are literature reports that *P. aeruginosa* could enhance its environmental stress tolerance under SMG through the AlgU → Hfq → Anr → HemN pathway [13]. But the *hfq*–*hemN* pathway has not been studied in *E. coli*. In this study, we found that *hemN* was positively regulated by *hfq*, and SMG could increase *hemN* expression. It was indicated that the *hfq*–*hemN* pathway might play an important role in oxidative stress tolerance of *E. coli* under SMG.

*rpoS* is a *σ* factor (*σ*^s^) in the stable growth stage of *E. coli* and a major regulator of most stress responses of *E. coli* [22]. *rpoS* could resist hypertonic stress and low- or high-temperature stress [21]. Previous studies have shown that *hfq* could regulate the metabolism of *E. coli* and produce positive regulation of *rpoS* to regulate the bacterial response to environmental stress [22, 25, 26]. But in this study, we found that the expression of *rpoS* was not regulated by *hfq* in *E. coli*, under both the NG and the SMG. But *rpoS* still could be affected by SMG, and the tolerance to hypertonic and acidic environments was enhanced, suggesting that changes in *E. coli* were not affected by the *hfq*–*rpoS* pathway in tolerance to hypertonic and acidic environmental stresses. It was also reported that *hfq* worked synergistically with *rpoS* to promote urethral pathogenic *E. coli* to resist and adapt to hostile host environments [43]. Therefore, we speculate that *hfq* and *rpoS* may be synergistic rather than upstream and downstream in hypertonic and acidic stress tolerance of *E. coli* under SMG.

This study preliminarily elucidated the effect of *hfq* on environmental tolerance of *E. coli* under SMG and its relationship with *hemN* and *rpoS*. In the further study, we will construct gene knockout strains of *hemN* and *rpoS* to verify the mechanism of interaction between *hfq* and *hemN* and *rpoS*. SMG-mutated *E. coli* will be used to infect host cells to explore the effect and mechanism of space mutagenesis *E. coli* on host cells.

## Figures and Tables

**Table 1:** Summary of test results.

**Number**	**Sample name**	**Density (ng/*μ*L)**	**Volume**	**Total (ng)**	**OD value**	**Test conclusion**
1	YJ	1860	100	186,000.0	1.999	A
2	SPY	1314	100	131,400.0	1.968	A
3	Gravite	1560	30	46,800.0	1.958	B

*Note:* YJ qualified; SPY qualified; Gravite can try building a database.

**Table 2:** Primer sequence of RT-qPCR (sort alphabetically).

**Primer**	**Primer sequence 5**⁣′**→3**⁣′
hemN-F	AACCAGAAAGAGTTGAAGCA
hemN-R	GTTTCTCAATAGGGGCGTAA
hfq-F	AACGTGTTCCAGTTTCTAT
hfq-R	ATGGTAGTTACTGCTGGTA
rpoS-F	GCCGTATGCTTCGTCTTAAC [10]
rpoS-R	GTCATCTTGCGTGGTATCTTC [10]
rrsB-F (16S)	GCATAACGTCGCAAGACCAAA [35]
rrsB-R (16S)	GCCGTTACCCCACCTACTAGCT [35]

**Table 3:** Environment tolerance-related genes in transcriptome sequencing analysis between ECG and ECN (sorted by gene ID).

**Gene ID**	**Name**	**Mean TPM (ECG)**	**Mean TPM (ECN)**	|**log**_2_ **fold** **change**|	**q** ** value**	**Result**
b2741	*rpoS*	1226.28	866.65	0.500767	5.99e − 60	+
b3867	*hemN*	467.54	430.92	0.11767	0.007464283	+
b4172	*hfq*	887.39	696.45	0.349549	9.87e − 08	+

*Note:* “+” indicates that the simulated recombinant gene is upregulated compared with the normal gravity group.

## Data Availability

The data that support the findings of this study are available from the corresponding authors upon reasonable request.
